# Cloud Follow-Up in Patients With Cardiovascular Implantable Electronic Devices: A Single-Region Study in China

**DOI:** 10.3389/fcvm.2022.864398

**Published:** 2022-05-09

**Authors:** Lin Tong, Shiqiang Xiong, Jun Hou, Jin Li, Shujuan Qin, Yangchun Zhang, Siqi Yang, Lingyao Qi, Xu Chen, Yan Luo, Zhen Zhang, Haoyu Deng, Hanxiong Liu, Lin Cai

**Affiliations:** ^1^Department of Cardiology, The Third People’s Hospital of Chengdu, Affiliated Hospital of Southwest Jiaotong University, Chengdu, China; ^2^Department of Medicine, Faculty of Medicine, University of British Columbia, Vancouver, BC, Canada; ^3^Centre for Heart and Lung Innovation, St. Paul’s Hospital, University of British Columbia, Vancouver, BC, Canada; ^4^Department of Vascular Surgery, Renji Hospital, School of Medicine, Shanghai Jiao Tong University, Shanghai, China

**Keywords:** cardiac implantable electronic device (CIED), in-office visit, follow-up, remote programming, telemedicine, COVID-19, remote interrogation, remote testing

## Abstract

**Background:**

Due to seriously imbalanced distribution of follow-up clinics in China, routine in-office visits are erratically attended by many cardiovascular implantable electronic device (CIED) patients. Meanwhile, remote monitoring is significantly underutilized. Novel tools to address the current predicament of routine in-office visits in China is urgently needed.

**Objectives:**

To assess the reliability and feasibility of cloud follow-up in CIED patients.

**Methods:**

A total of 325 CIED patients from 13 hospitals in Sichuan Province, China, were enrolled. Information on patients’ sociodemographic and basic clinical characteristics was collected. All devices were tested and programmed with 5G-cloud follow-up platform in a real-time manner. All patients were surveyed about their acceptance of and preferences regarding cloud follow-up compared to routine in-office visits.

**Results:**

Compliance with routine in-office visits in this region was 60.6%. None of the patients were enrolled in remote monitoring services. Clinically important predictors of non-compliance were elderly age (≥75 years old), odds ratio (OR) 2.392 (95% confidence interval, 1.111–5.150); needing notification from a follow-up clinic, OR 2.518 (1.179–5.376); and being beyond 15 months post-implantation, OR 5.440 (2.563–11.543). All cloud follow-up sessions were performed safely and efficiently, without any adverse events. 292 (89.8%) patients preferred cloud follow-up for future device management.

**Conclusion:**

Compliance with routine in-office visits in this region has much room for improvement. Cloud follow-up addresses the limitations of an imbalanced distribution of follow-up clinics and geographic barriers for in-office CIED evaluation. Thus, cloud follow-up provides a potential solution to the current predicament of routine in-office visits in China.

## Introduction

Postimplantation follow-up of patients with cardiac implantable electronic devices (CIEDs) is crucial for monitoring device function and improving patient outcomes (1). In China, since 2018, more than one million CIED patients require follow-up every year (2). However, at present most patients cannot participate in all of the scheduled in-office visits for long-term CIED management (2). Both limited follow-up clinic resources and geographical distance are important barriers to completing in-office CIED evaluations. The outbreak of the COVID-19 pandemic further induced a drastic reduction in the frequency of in-office visits and acceleration of the development of telemedicine (3–5). Remote monitoring (RM) of CIEDs has emerged as an important tool for optimizing the use of limited medical resources and providing safe and efficient after-care service to CIED patients. However, due to the lack of reimbursement and logistics to ensure smooth operation of the closed-loop management involved in RM, RM is significantly underused in China. Less than 10% of permanent pacemakers (PPMs) patients are enrolled in RM follow-up services for their devices (2, 6). Therefore, novel complementary tools are urgently needed to replace some of the routine in-office visits.

As remote technology has developed rapidly, remote programming (RP) of CIEDs has, unsurprisingly, been explored. In 2019, the first clinical application of real-time RP of CIEDs was shown to be feasible, safe, and clinically relevant in a magnetic resonance imaging setting (7). Our team has performed remote testing and programming of CIEDs by using a 5G-cloud follow-up platform during the device implantation procedure (8). In response to the COVID-19 pandemic, Okabe et al. implemented RP of CIEDs with the goal of minimizing personnel exposure to COVID-19 infection (9). Moreover, amid the pandemic, the use of telemedicine has been encouraged in most circumstances to protect patients and health care teams from COVID-19 exposure (10). With judicious clinical use of RP, the technical reliability and feasibility of RP is in urgent need of verification in broader clinical practice.

In consideration of the present hurdles for in-office visits and RM in China, top-tier hospitals could provide real-time RP-centered cloud follow-up services for primary care institutions in areas lacking follow-up clinics to improve the management of follow-up in CIED patients and minimize the risk of cross infection during the COVID-19 pandemic. As no precedent exists, we focused our investigation on this issue to evaluate the reliability and feasibility of cloud follow-up in CIED patients. In addition, we explored the satisfaction of the study population and their acceptance of cloud follow-up.

## Materials and Methods

### Study Design and Patients

The present study is a multicenter, observational trial registered in Chinese Clinical Trial Registry (ChiCTR2100046883). The study conducted in 13 hospitals (the regional medical consortiums of The Third People’s Hospital of Chengdu) in Sichuan Province of China was designed to evaluate the technical reliability and feasibility of real-time RP- centered follow-up in CIED patients. The study complied with the Declaration of the Helsinki with respect to investigation in humans. The appropriate institutional review board committees of each participating center approved the protocol of present study and written informed consent was obtained from all patients. The enrollment of patients began in May 2021 and ended in December 2021.

We included only patients with St. Jude CIEDs-that are compatible with the cloud follow-up system (Abbott Laboratories, North Chicago, Illinois, United States). The only selection criteria were that patients or their caretakers were able to complete the questionnaires and willing to receive cloud follow-up. Information on patients’ sociodemographic and basic clinical characteristics was obtained *via* the baseline questionnaire. Information on patients’ CIEDs, and comorbidities was extracted from their medical records, and entered into an electronic case report form by the investigators of the research team. In the questionnaire, patients were also asked about participation in routine in-office visits. Finally, all patients completed a set of follow-up questionnaires at the end of the cloud follow-up. The questionnaire surveyed their preferences and satisfaction regarding cloud follow-up compared to routine in-office visits and whether cloud follow-up had a positive effect on their peace of mind.

The study group comprised 295 patients with PPMs, 16 with implantable cardioverter defibrillators (ICDs), 9 with ICD-cardiac resynchronization therapy (CRT-D), and 5 with CRT- pacemakers (CRT-Ps). A Merlin Patient Care System Programmer Model 3,650 (St. Jude Medical Inc., Saint Paul, Minnesota, United States) was activated and the wand was placed in a conventional manner by the local medical staff, while the device specialist (an electrophysiologist) performed real-time remote testing and RP in the CIED Follow-up Center of The Third People’s Hospital of Chengdu.

### The 5G-Cloud Follow-Up Platform and Communications Protocol

**FIGURE 1:**
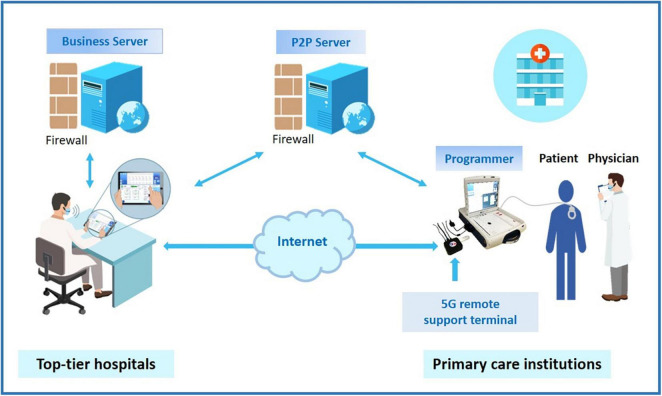
The schematic of application of 5G-cloud follow-up. Top-tier hospitals provide cloud follow-up services for primary care institutions in areas lacking follow-up clinics. By addressing the limitations of an imbalanced distribution of follow-up clinics and geographic barriers, cloud follow-up makes in-office visits more accessible for CIED patients living in remote areas. Moreover, cloud follow-up is particularly convenient for patients with a great need for urgent device programming. CIED, cardiovascular implantable electronic device.

This study employed a 5G-cloud follow-up platform (China Telecom Corporation Limited Shanghai Branch, Shanghai), a research tool that allows a device specialist to test, and program CIEDs in real-time from a remote location *via* an internet connection or mobile wireless network (Figure 1). Consistent with of the 5G remote support terminal (China Telecom Corporation Limited Shanghai Branch, Shanghai) externally connected to the programmer, a PAD was installed with a 5G-cloud follow-up application, and the whole remote service system was deployed on a cloud server with rigorous security protections including multilayer firewalls, customized antivirus scanning, vulnerability scanning and intrusion detection to ensure user data security (Figure 1).

The 5G-cloud follow-up platform has rigorous security procedures to authenticate and protect the connection. The onsite medical staff, after obtaining written informed consent from the patient, began the cloud follow-up session by contacting the remote device specialist *via* video call. The connection to the CIED was made by the medical staff through the use of a standard programmer. He was in charge of turning on the programmer and applying the programmer wand to the patient’s device. After an initial introduction, communication was established and continued *via* the wand connection. The remote device specialist logged into the 5G-cloud follow-up application on a PAD with two-step verification: Step 1: log into the designated account using a password, and Step 2: use the access password for the second verification to establish remote connection for the designated device. The remote device specialist then had complete control of the programmer functions to check and reprogram the device as needed. Asymmetric cryptographic algorithms, sophisticated end-to-end secure communication protocols, and private cloud deployment were used to protect the cybersecurity of the information and communications. When communication between the on-site programmer and the remote electrophysiologist’s PAD is interrupted, the device will revert to the original settings. The whole remote process can be saved *via* screen recording. The log documents the start and end times, duration, and function modules used for each remote operation, allowing users to easily audit the logs and statistics later.

A precheck of the system to assure reliable connectivity before each session is an important security arrangement to protect patient safety. During each cloud follow-up session, the onsite medical staffs will provide medical assistance to patients, and communicate with the remote electrophysiologist by using the audio and video communication function of mobile phones. In order to observe the remote scene clearly, we displayed the contents of the mobile phone screen on an external large monitor. The ability to perform first aid and troubleshoot occasionally arising technical issues is essential for the onset medical staffs.

### Outcome Measures and Statistical Analysis

All study data were collected and recorded by the investigators of the research team on the case record form. Compliance with in-office visits was defined according to the Chinese Society of Pacing and Electrophysiology Guidelines (11). The first post-discharge in-office visit should be performed during 4–12 weeks post-implantation. In-office visit should be planned every 3–12 months thereafter depending on the patient’s clinical condition and the type of CIED. ICD/CRT follow-up should usually occur at no longer than 6-month intervals. For cloud follow-up sessions, outcome measures included the completion of successful communications protocols and successful remote follow-up and management of the CIEDs, with time measurements obtained for efficiency. Continuous variables are described by means, and standard deviations (SDs); and categorical variables are expressed as counts and percentages in each category. A logistic regression model was used to determine odds ratios (ORs) of non-compliance. A *p*-value less than 0.05 was considered statistically significant. All analyses were performed using either GraphPad Prism software (version 8.0, GraphPad Software, San Diego, CA, United States) or SPSS (version 19.0, SPSS Inc., Chicago, Il, United States).

## Results

### Baseline Characteristics of Cardiovascular Implantable Electronic Device Patients and Their Compliance With Routine In-Office Visits

**FIGURE 2:**
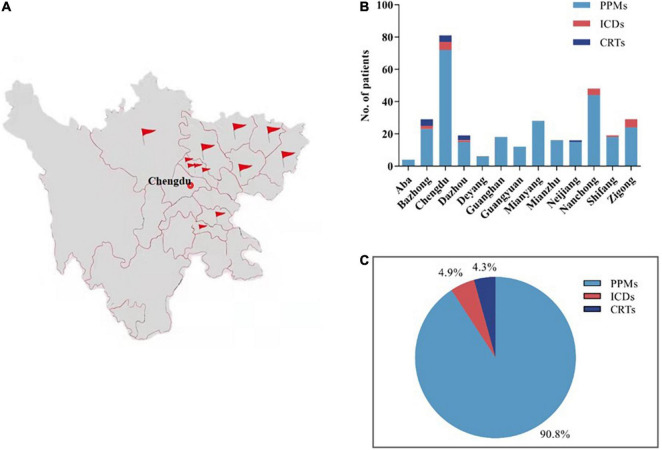
The general profile of the present study. A total of 325 patients from 13 hospitals in Sichuan, China, were enrolled in this study **(A,B)**, including 295 (90.8%) patients with PPMs, 16 (4.9%) patients with ICDs and 14 (4.3%) patients with CRTs **(C)**. PPMs, permanent pacemakers; ICDs, implantable cardioverter defibrillators; CRTs, cardiac resynchronization therapies.

The participating hospitals widely distributed throughout Sichuan Province (Figure 2A). A total of 325 patients from 13 hospitals took part in this study, including 295 (90.8%) patients with PPMs, 16 (4.9%) patients with ICDs and 14 (4.3%) patients with CRTs (Figures 2B,C). None of the patients were enrolled in RM services for their devices. These results are quite different from other studied CIED populations from Western countries, or even developed Asian countries or regions (12–15).

The baseline characteristics of the study population are presented in Table 1. The mean age was 73.6 ± 10.7 years and 172 (52.9%) were elderly people (≥75 years of age). 165 (50.8%) were females, and 320 (98.5) had medical insurance. Coronary artery disease [115 (35.4%)], hypertension [108 (33.2%)], atrial fibrillation/flutter [83 (25.5%)] and diabetes mellitus [48 (14.8%)] were the most common comorbidities.

**TABLE 1:** Baseline characteristics of patients from each cardiac implantable electronic device groups.

Characteristic	Total (325)	PPMs (*n* = 295)	ICDs (*n* = 16)	CRTs (*n* = 14)
**Sociodemographic characteristics**				
Age, mean ± *SD*, y	73.6 ± 10.7	73.9 ± 10.8	69.6 ± 10.4	70.7 ± 7.9
<75 y, No. (%)	153 (47.1)	133 (45.1)	11 (68.8)	9 (64.3)
≥75 y, No. (%)	172 (52.9)	162 (54.9)	5 (31.3)	5 (35.7)
Female	165 (50.8)	156 (52.9)	4 (25.0)	5 (35.7)
Living alone	53 (16.3)	52 (17.6)	0 (0)	1 (7.1)
Medical insurance—No. (%)	320 (98.5)	290 (98.3)	16 (100)	14 (100)
Senior Secondary Education or higher—No. (%)	62 (19.1)	60 (20.3)	1 (6.3)	1 (7.1)
**Life style—No. (%)**				
Body mass index (kg/m^2^), mean ± *SD*	23.2 ± 3.4	23.2 ± 3.7	23.6 ± 3.1	21.9 ± 2.6
Smoking	34 (10.5)	29 (9.8)	3 (18.8)	2 (14.3)
Use of alcohol	35 (9.7)	29 (10.6)	3 (18.8)	2 (14.3)
**Systolic BP, mean ± *SD*, mmHg**	139.1 ± 21.0	140.0 ± 20.9	128.4 ± 19.7	132.5 ± 21.9
**Diastolic BP, mean ± *SD*, mmHg**	81.2 ± 13.2	81.7 ± 13.2	75.9 ± 12.0	78.2 ± 14.3
**Heart rate, mean ± *SD*, bpm**	69.2 ± 12.0	69.1 ± 12.0	67.7 ± 12.9	72.3 ± 11.2
**Comorbidity**				
Coronary artery disease—No. (%)	115 (35.4)	104 (35.3)	8 (50.0)	3 (21.4)
Hypertension—No. (%)	108 (33.2)	102 (34.6)	5 (31.3)	1 (7.1)
Diabetes mellitus—No. (%)	48 (14.8)	46 (15.6)	1 (6.3)	1 (7.1)
Dyslipidemia—No. (%)	16 (4.9)	10 (4.8)	3 (18.8)	3 (21.4)
Dilated cardiomyopathy—No. (%)	13 (4.0)	−	5 (31.3)	8 (57.1)
Atrial fibrillation/flutter—No. (%)	83 (25.5)	75 (25.4)	4 (25.0)	4 (28.6)
Stroke—No. (%)	13 (4.0)	13 (4.4)	−	−
**Implant indications**				
Sick sinus syndrome—No. (%)	−	114 (38.6)	−	−
2nd/3rd degree AV block—No. (%)	−	110 (37.3)	−	−
Primary prevention—No. (%)	−	−	10 (62.5)	3 (21.4)
Secondary prevention—No. (%)	−	−	6 (37.5)	2 (14.3)
Other—No. (%)	−	71 (24.1)	−	9 (64.3)
**Appointments of in-office visits**				
Initiative visits—No. (%)	200 (61.5)	178 (60.3)	10 (62.5)	12 (85.7)
Notifications from follow-up clinics—No. (%)	125 (38.5)	117 (39.7)	6 (37.5)	2 (14.3)
**Transportation**				
On foot	41 (12.6)	39 (13.2)	1 (6.3)	1 (7.1)
Car	100 (30.8)	88 (29.8)	4 (25.0)	8 (57.1)
Public transport means	184 (56.6)	168 (56.9)	11 (68.8)	5 (35.7)
Having a companion	227 (69.8)	199 (67.5)	16 (100)	12 (85.7)
Travel time, mean ± *SD*, min	36.5 ± 32.9	39.2 ± 57.0	36.3 ± 28.7	37.7 ± 29.8
**Compliance with routine in-office visits**	197 (60.6)	174 (59.0)	12 (75.0)	11 (78.6)
The first visit 4–12 weeks post-implantation	229 (70.5)	204 (69.2)	13 (81.3)	12 (85.7)
Every 3–12 months for PPMs	−	161/240 (67.1)	−	−
Every 3–6 months for ICDs and CRTs	−	−	7/10 (70.0)	8/10 (80.0)

*Data are presented as mean ± SD, or No. (%). PPMs, permanent pacemakers; ICDs, implantable cardioverter defibrillators; CRTs, cardiac resynchronization therapies.*

61.5% of the CIED patients completed routine in-office visits spontaneously (Table 1). Meanwhile, 38.5% of the CIED patients did not attend visits until they had received notifications from the follow-up clinics. The overall compliance with in-office visits in this region was 60.6%. 70.5% of CIED patients completed routine in-office visits within 4–12 weeks post-implantation, and this percentage is much higher than that in a previous report (2).

### Factors Associated With Non-compliance With Routine In-Office Visits

**FIGURE 3:**
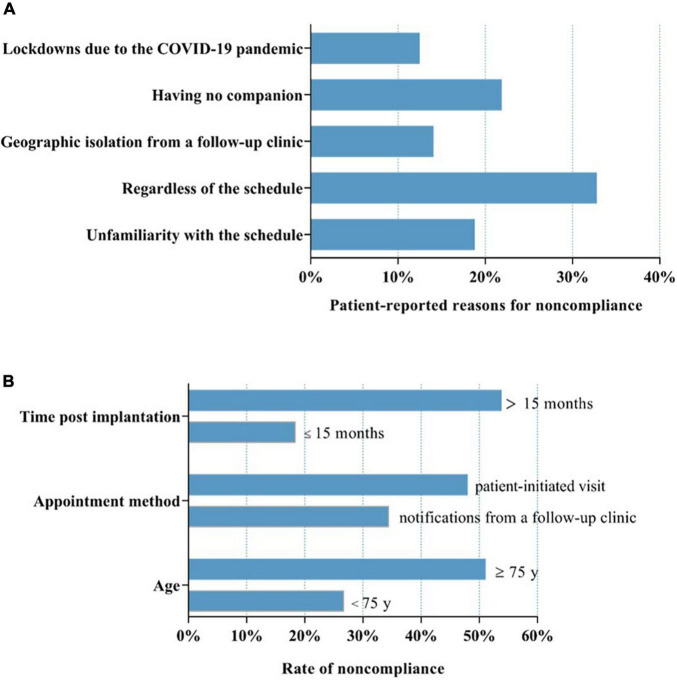
Factors associated with non-compliance with routine in-office visits. **(A)** Patients who displayed non-compliance with routine in-office visits reported that regardless of the schedule because of no discomfort (32.8%), having no companion (21.9%), lack of familiarity with the follow-up schedule (18.8%), geographic isolation from the follow-up clinics (14.1%) and lockdowns due to the COVID-19 pandemic (12.5%) resulted in non-compliance. **(B)** Factors including age, appointment method and time post-CIED implantation were significantly associated with variations in non-compliance. COVID-19, coronavirus disease 2019. “Disregardness of the schedule” indicates the patients did not take routine in-office visit positively, and refused to finish it on time or even did not attend it. “Unfamiliarity with the schedule” means the patients were unfamiliar with the in-office visit arrangements, which made them fail in finishing the in-office visit on time or even miss it.

128 (39.4%) patients who displayed non-compliance with routine in-office visits reported that regardless of the schedule because of no discomfort (32.8%) contributed to their non-compliance and that having no companion (21.9%), lack of familiarity with the follow-up schedule (18.8%), geographic isolation from the follow-up clinics (14.1%) and lockdowns due to the COVID-19 pandemic (12.5%) also contributed to non-compliance (Figure 3A).

In this study population, factors including age, appointment method and time post-CIED implantation were significantly associated with variations in non-compliance. Compared with patients < 75 years of age, patients ≥ 75 years of age had a higher rate of non-compliance (51.1 vs. 26.8%, *P* < 0.01; Figure 3B). The patients who completed the routine in-office visits spontaneously without the need for notification from a follow-up clinic exhibited a lower rate of non-compliance (34.5 vs. 48.0%, *P* < 0.05; Figure 3B). Patients had a greater rate of compliance within 15 months post-CIED implantation (18.5 vs. 53.8%, *P* < 0.0001; Figure 3B). However, sex, device type, education level and having a companion had no significant effect on the rate of non-compliance.

Logistic regression analysis with respect to the variables we studied showed that age, appointment method and time post-CIED implantation were all statistically significant independent predictors of non-compliance (Table 2). In the adjusted analysis, elderly individuals (≥75 years old) were 2.4 times more likely to be non-compliant than younger patients (<75 years old; 95% CI: 1.1–5.2), *P* < 0.05. Non-compliance was 2.5 times more likely in patients needing notification from a follow-up clinic (95% CI: 1.2–5.4), *P* < 0.05. The odds of non-compliance were 5.4 times as high in patients who had undergone CIED implantation more than 15 months as those who had undergone CIED implantation within the past 15 months (95% CI: 2.6–11.5), *P* < 0.001.

**TABLE 2:** Odds ratios of non-compliance.

	OR	95% CI	*p*-value
Age ≥ 75 vs. < 75 years old	2.4	1.1–5.2	0.026
Notifications from a follow-up clinic vs. patient-initiated visit	2.5	1.2–5.4	0.017
Time post-implantation ≥ 15 months vs. < 15 months	5.4	2.6–11.5	<0.001

*OR, Odds Ratio; CI, confidence interval.*

### The Results of In-Time Remote Interrogation, Testing and Programming of Cardiovascular Implantable Electronic Devices

A total of 325 devices was interrogated, tested and programmed with 5G-cloud follow-up. The average duration for cloud follow-up was 5.4 ± 3.5 min. This time included device checks, reprogramming and brief communication among the patient, onsite medical stall and remote device specialist. The alert messages for CIEDs in the study group are shown in Table 3.

**TABLE 3:** Alert messages for each cardiac implantable electronic device groups.

Items	Events (*n*)
**PPMs**	
*Device*	
Elective replacement indicator	1
*Leads*	
Right atrial impedance > 2,000 Ω	1
The atrial sensitivity < 2:1 safety margin	7
Right ventricular impedance > 2,000 Ω	1
The ventricular sensitivity < 2:1 safety margin	7
R-wave amplitude < 2:1 safety margin	4
*Diagnostic*	
High percentage of ventricular pacing	2
Excessive burden of AF/AF	7
Pacemaker mediated tachycardia	26
High ventricular rate	14
Atrial noise reversion of paroxysmal atrial fibrillation	3
Reprogramming n/N (%)	94/295 (31.9)
**ICDs**	
*Device*	
Elective replacement indicator	0
*Diagnostic*	
High percentage of ventricular pacing	7
Excessive burden of AF/AF	2
Supraventricular tachycardia	1
Atrial noise reversion	1
*Fast ventricular rate*	
Pacemaker mediated tachycardia	2
Magnet reaction	1
ST-segment alteration event (Class I)	1
Reprogramming n/N (%)	4/16 (25.0)
**CRTs**	
*Device*	
Elective replacement indicator	0
Diagnostic	
Low biventricular pacing percentage	3
Excessive burden of AF/AF	4
*VT/VF events more than 3 times*	
Magnet reaction	1
Fast ventricular rate	2
Atrial noise reversion	2
Reprogramming n/N (%)	5/14 (35.8)

*AF/AF, atrial tachycardia and atrial fibrillation; VT, ventricular tachycardia and atrial fibrillation. PPMs, permanent pacemakers; ICDs, implantable cardioverter defibrillators; CRTs, cardiac resynchronization therapies.*

The most common alert messages in PPM patients were pacemaker-mediated tachycardia (26/295), a high ventricular rate (14/295), low ventricular sensitivity (7/295), and an excessive burden of atrial tachycardia and atrial fibrillation (7/295). An elective replacement indicator was activated in 1 PPM patient. The most frequent alert in ICD patients was a “high percentage of ventricular pacing” (7/16) and “excessive burden of atrial tachycardia and atrial fibrillation” (4/14) was the most frequent alert in CRT patients.

94 (31.9%) patients with PPMs, 4 (25.0%) with ICDs, and 5 (35.8%) with CRTs were reprogrammed remotely as appropriate (Table 4). Ventricular autocapture, atrioventricular intervals, and ventricular intrinsic preference were the most common adjustments in PPM devices. Reprogrammed items in ICD devices consisted of ACap™ Confirm, hysteresis mode, cycle length for VF zone, and base rate. Adjustment of bi-ventricular auto-capture or interventricular intervals was relatively common in CRT devices. The connectivity was adequate and there were no errors about communication during the cloud follow-up sessions. An occasional transmission delay, estimated to be a maximum of 3 s, was not found to be clinically significant, and thus did not detract from providing adequate patient care. No complications or adverse events occurred, and there was no need for the backup physician to intervene during the study.

**TABLE 4:** Remote reprogramming performed in each cardiac implantable electronic device groups.

Items	Reprogramming (*n*)
**PPMs**	
Ventricular AutoCapture	Off→On (25), On→Off (1)
ACap™ confirm	Off→On (1)
Ventricular intrinsic preference	Off→On (23)
Pacing modes	Mode adjustment (13)
Hysteresis mode	Off→On (13), Parameter adjustment (2)
Atrial pulse amplitude	Parameter adjustment (2)
Ventricular pulse amplitude	Parameter adjustment (1)
Atrioventricular intervals	Parameter adjustment (25)
Lead polarity	Parameter adjustment (15)
Upper tracking rate	Parameter adjustment (3)
Rest rate	Parameter adjustment (1)
**ICDs**	
ACap™ Confirm	Off→On (1)
Hysteresis mode	Off→On (1)
Base rate	Parameter adjustment (1)
Cycle length for VF zone	Parameter adjustment (1)
**CRTs**	
ACap™ confirm	Off→On (1)
Bi-ventricular AutoCapture	Off→On (1)
Atrioventricular intervals	Parameter adjustment (2)
Interventricular intervals	Parameter adjustment (2)
Hysteresis mode	Off→On (1)

*PPMs, permanent pacemakers; ICDs, implantable cardioverter defibrillators; CRTs, cardiac resynchronization therapies; VF, ventricular fibrillation.*

### Patient-Reported Acceptance of and Preference for Cloud Follow-Up for Cardiovascular Implantable Electronic Devices

All patients enrolled in this study completed the questionnaire (Table 5). 97.8% (318) of the patients trusted cloud follow-up and 86.5% (281) of the patients did not feel uneasy throughout cloud follow-up. Only 33 patients (10.2%) preferred in-person evaluation to cloud follow-up, and 292 (89.8%) patients chose cloud follow-up for further device management. The preference for more human interaction was the primary reason for choosing in-person evaluation. The level of acceptance of and preference for cloud follow-up seem to be higher in ICD and CRT patients.

**TABLE 5:** Patient questionnaire results.

Response	Total (*n* = 325)	No. (%)		
		PPMs (*n* = 295)	ICDs (*n* = 16)	CRTs (*n* = 14)
**Do you feel uneasy with your device having been remotely evaluated and reprogrammed instead of in-person evaluation?**				
Yes, very uneasy	6 (1.8)	4 (1.4)	1 (6.3)	1 (7.1)
Somewhat uneasy	38 (11.7)	38 (12.9)	0 (0)	0 (0)
No	281 (86.5)	253 (85.7)	15 (93.8)	13 (92.9)
**Do you trust cloud follow-up?**				
Yes	318 (97.8)	290 (98.3)	15 (93.8)	13 (92.9)
No	7 (2.2)	5 (1.7)	1 (6.3)	1 (7.1)
**Would you choose cloud follow-up in future device management?**				
Yes	292 (89.8)	264 (89.5)	15 (93.8)	13 (92.9)
No	33 (10.2)	31 (10.5)	1 (6.3)	1 (7.1)

*PPMs, permanent pacemakers; ICDs, implantable cardioverter defibrillators; CRTs, cardiac resynchronization therapies.*

## Discussion

Our study investigated the reliability and feasibility of cloud follow-up in CIED patients. The overall compliance with in-office visits in this single-region study was 60.6%, demonstrating significant room for improvement. Factors including age, appointment method and time post-CIED implantation were all statistically significant independent predictors of non-compliance. All cloud follow-up sessions were performed safely and efficiently, without any complications or adverse events. Most CIED patients were very trusting and preferred cloud follow-up for future device management. Cloud follow-up may be a novel service model for improving CIED follow-up management in patients living in remote areas lacking follow-up clinics. In the current COVID-19 crisis, cloud follow-up should be used in most circumstances to minimize personnel exposure to COVID-19 infection.

In this single-region multicenter study, a total of 325 patients participated, with 295 (90.8%) patients with PPMs, 16 (4.9%) patients with ICDs and 14 (4.3%) patients with CRTs. The proportion of ICDs and CRTs was much lower than previous reports from Western countries and well-developed Asian regions (12–16). The causes of such differences are multifactorial, including differences in disease patterns, patient acceptance, cost, and reimbursement. Unfortunately, because of data limitations, we were unable to obtain accurate estimates of the implantation rates of different device types in this region. As the indications for implantation broaden and the frequency of device utilization increases, the management of these patients and their devices has become increasingly complex and important. The overall compliance with in-office visits in this study was 60.6%., which is similar to the result of a previous registry study in mainland China (17). Because China is vast in territory, patients are often geographically distant from their implanting and monitoring centers, and in-office visits are a burden and erratically attended by many patients (17). Thus, geographical barriers have become a common limitation for in-office visits.

As is common in all countries, the distance to and logistics for reaching a clinic for in-office visits are closely associated to the compliance rate (18). Among patient factors, age was a significant predictor of non-compliance. Elderly patients were more likely to be non-compliant with in-office visits. We found that patients needing notification from a follow-up clinic and being more than 15 months post-CIED implantation were also independent predictors of non-compliance. Patient-reported reasons for non-compliance included unfamiliarity with the follow-up schedule, regardless of the schedule because of no discomfort, geographic isolation from follow-up clinics, lockdowns due to the COVID-19 pandemic and having no companion. These results indicate that both active patient participation post-device implantation and patient education are essential for in-office visits to ensure greater patient retention. Furthermore, the COVID-19 outbreak has had a profound impact on the organization of health care related to arrhythmias and electrophysiology (3, 10). During the pandemic, where possible, in-person clinical evaluation should be avoided (10). There is no doubt that the postponement and cancelation of appointments, and limitations on visitors in follow-up clinics during the pandemic resulted in a higher rate of non-compliance with in-office visits in this study population. In light of the advantages of RM largely eliminating the need for active patient participation in in-office visits, RM is an important tool for ensuring greater patient retention (1). When feasible, the routine use of RM in CIED management post-implantation has been encouraged (10). However, RM is significantly underused due to cost, lack of reimbursement, and logistic support (2, 6, 17). For example, the percentage of PPMs with RM service was less than 10% in mainland China (2, 6). In the present study, none of the CIED patients were enrolled in RM services for their devices. The management of CIED follow-up varies among geographic locations and socioeconomic and medical structures, but the goal is to provide standard follow-up service for the whole CIED population.

Nevertheless, there is a great demand for remote patient management in China. Our successful application of cloud follow-up in the clinic depended on the wide availability of 5G networks in China. The use of a 5G network enables the transfer of large amounts of data at fast rates. It allows a device specialist to interrogate, test, and program CIEDs in real-time from anywhere he has access to an internet connections or mobile wireless networks. No communication problems occurred during the cloud follow-up sessions, and all alert settings were reprogrammed remotely as appropriate. Most patients were very trusting and preferred cloud follow-up for future device management. Few previous studies have examined real-time RM of CIEDs in other clinical sessions, including magnetic resonance imaging scans (7), and device implantation (8, 9). Consistent with others’ results, remote management of CIEDs has been proven to be safe, efficient, and feasible. The implementation of cloud follow-up has great potential help address the imbalanced distribution of follow-up clinics and make geographic barriers no longer a hurdle for in-person CIED evaluation. In the current COVID-19 crisis, cloud follow-up is beneficial in reducing transregional transportation, thus minimizing personnel exposure to COVID-19 infection. Moreover, cloud follow-up is particularly convenient for patients with a great need for urgent device programming.

Patient safety is the major concern in cloud follow-up sessions, especially in capture tests. In the early stage of this study, there were occasional transmission delays, estimated to be a maximum of 3 s, was not found to be clinically significant. This kind of communication problems were remarkedly improved through updating the firmware of 5G remote support terminal. Transmission delay occurring in a capture test will result in the interruption of the continuous signal received by the programmer, which assumes that the stylus/finger have left the testing button. Thus, the occurrence of transmission delay will terminate the capture test automatically and revert the device to the original settings. In case of capture lost in a pacemaker-dependent patient, which may also happen in routine capture test, the onsite medical staff should immediately reprogram the device to emergency VVI pacing for bradycardia by activating the emergency pacing switch located on the programmer. After the connectivity is restored, the remote device specialist may continue to check the device. The remote device specialist will suspend the cloud follow-up session and check the patient personally if necessary.

In recent years, medical devices including CIEDs have become increasingly interconnected. There is no doubt that this added interconnectivity has already provided substantial benefit to CIED patients with RM. Meanwhile, this progress in technology comes with new challenges, including cybersecurity (19). Cybersecurity vulnerability is currently considered as a high priority, which challenges the ability of healthcare organizations to provide adequate care, the same with cloud follow up. As security vulnerabilities exist in all software, judicious and appropriate use of the remote programming technology is needed before we gain more experience in clinical settings. In case of remotely programing a CIED device, clinicians should weigh the potential risks about safety and cybersecurity vulnerability. Meanwhile, we propose that cloud follow-up should be treated like any other situation in which communications with sensitive material occur in cyberspace, such as RM, banking, air traffic control and military communications (7). In this study, we applied several layers of security to protect the safety of both the patients and cybersecurity. We should acknowledge that the world has changed and that the collective CIED community needs to rise to and meet new challenges (19). Cybersecurity is the responsibility of all stakeholders (e.g., industry, health care providers, government) and requires increased collaboration and communication across the community as the landscape continues to evolve (19, 20).

## Limitations

The present study was designed to be a proof-of-concept and feasibility investigation in a real clinical setting; as such, it was a single-arm, non-randomized study. Prospective randomized controlled studies addressing compliance and clinical outcomes with cloud follow-up will be critical. Our study was limited by the small number of patients implemented with ICD or CRT devices. Thus, there is a need for large prospective studies using a rigorous study protocol. Our definition of compliance, although based on a variety of guidelines, is somewhat rigid and was designed specifically to dichotomize patients and provide information about longitudinal follow-up. Unfortunately, some patients who needed notification from a follow-up clinic were classified as non-compliant because of an overdue appointment with a follow-up clinic. Although there is currently only one CIED vendor with equipment that can readily perform cloud follow-up (St. Jude Medical Inc., Saint Paul, Minnesota, United States), the general concept and service model are applicable to all vendors.

## Conclusion

The compliance with routine in-office visits in this single-region study was 60.6%, suggesting that there is much room for improvement. Because the implementation of RM is rare at present, overcoming the problems linked to a lack of reimbursement or a lack of official general plans is crucial for large-scale implementation in the future. Cloud follow-up was efficient with no complications. The clinical application of cloud follow-up provides a solution to address the predicament of regular CIED follow-up in China. This innovative tool may has the potential to improve the management of routine in-office follow-up and the clinical prognosis in a certain group of CIED patients. Simultaneously, with judicious application of this tool, broader application in clinical settings may be possible, along with further development of CIED follow-up paradigms and protocols.

## Data Availability Statement

The original contributions presented in the study are included in the article/supplementary material, further inquiries can be directed to the corresponding authors.

## Ethics Statement

The studies involving human participants were reviewed and approved by the Third People’s Hospital of Chengdu. The patients/participants provided their written informed consent to participate in this study.

## Author Contributions

LT, SX, and JH were major contributors in the collection, analysis, interpretation of data, and drafting of the manuscript. JL, SQ, YZ, SY, LQ, XC, YL, and ZZ participated in performance of the realtime remote programming, the collection, and analysis of data. HD revised the manuscript for important intellectual content. HL and LC designed the study, had full access to all of the data in the study, and finally approved the manuscript submitted. All authors read and approved the final manuscript.

## Conflict of Interest

The authors declare that the research was conducted in the absence of any commercial or financial relationships that could be construed as a potential conflict of interest.

## Publisher’s Note

All claims expressed in this article are solely those of the authors and do not necessarily represent those of their affiliated organizations, or those of the publisher, the editors and the reviewers. Any product that may be evaluated in this article, or claim that may be made by its manufacturer, is not guaranteed or endorsed by the publisher.
